# Highly Immunogenic Nanoparticles Based on a Fusion Protein Comprising the M2e of Influenza A Virus and a Lipopeptide

**DOI:** 10.3390/v12101133

**Published:** 2020-10-06

**Authors:** Anna A. Zykova, Elena A. Blokhina, Roman Y. Kotlyarov, Liudmila A. Stepanova, Liudmila M. Tsybalova, Victor V. Kuprianov, Nikolai V. Ravin

**Affiliations:** 1 Institute of Bioengineering, Research Center of Biotechnology of the Russian Academy of Sciences, 119071 Moscow, Russia; nuta2109@gmail.com (A.A.Z.); blohina-lena87@mail.ru (E.A.B.); bercut@gmail.com (R.Y.K.); 2 Research Institute of Influenza, Russian Ministry of Health, 23805 St. Petersburg, Russia; stepanoval60@mail.ru (L.A.S.); sovet@influenza.spb.ru (L.M.T.)

**Keywords:** influenza, recombinant vaccine, M2e peptide, lipopeptide, nanoparticle

## Abstract

The highly conserved extracellular domain of the transmembrane protein M2 (M2e) of the influenza A virus is a promising target for the development of broad-spectrum vaccines. However, M2e is a poor immunogen by itself and must be linked to an appropriate carrier to induce an efficient immune response. In this study, we obtained recombinant mosaic proteins containing tandem copies of M2e fused to a lipopeptide from *Neisseria meningitidis* surface lipoprotein Ag473 and alpha-helical linkers and analyzed their immunogenicity. Six fusion proteins, comprising four or eight tandem copies of M2e flanked by alpha-helical linkers, lipopeptides, or a combination of both of these elements, were produced in *Escherichia coli*. The proteins, containing both alpha-helical linkers and lipopeptides at each side of M2e repeats, formed nanosized particles, but no particulate structures were observed in the absence of lipopeptides. Animal study results showed that proteins with lipopeptides induced strong M2e-specific antibody responses in the absence of external adjuvants compared to similar proteins without lipopeptides. Thus, the recombinant M2e-based proteins containing alpha-helical linkers and *N. meningitidis* lipopeptide sequences at the N- and C-termini of four or eight tandem copies of M2e peptide are promising vaccine candidates.

## 1. Introduction

Influenza A is a widely distributed respiratory infection of humans and animals. A high variability of major surface proteins, hemagglutinin and neuraminidase, results in the frequent appearance of new epidemic strains and appropriate renewal of conventional influenza vaccines [1]. In view of this limitation, the development of “universal” recombinant vaccines based on conserved influenza viral proteins is an important task [2,3]. Recombinant proteins containing conserved regions of viral proteins (hemagglutinin, neuraminidase, nucleoprotein, M1) and the extracellular domain of transmembrane protein M2 (M2e) are promising candidates for the development of a universal vaccine [4].

M2e is a 24 amino acid long (including the initiator methionine) peptide that is highly conserved in influenza strains of human origin and, thus, presents an attractive target for a “universal” influenza vaccine [5,6]. Although anti-M2e antibodies do not neutralize the virus, M2e-based vaccines can confer both humoral- and cell-mediated immune responses directed towards infected cells exposing M2e on the surface. M2e-specific protective antibodies act via antibody-dependent cell cytotoxicity and mediate the killing of infected cells by complement or natural killer cells [2]. In addition, anti-M2e antibodies bind the virus to the cell and prevent the release of viral particles or enhance the uptake by phagocytic cells via the Fc receptor [2].

Native M2e is a poor immunogen [7], but this problem can be solved by the fusion of M2e to special carriers and/or adjuvants that enhance its immunogenic properties. Virus-like nanoparticles formed by viral capsid proteins can be used as carriers of the M2e peptide [5,6,8,9,10,11]. In addition, bacterial flagellin, the major structural protein of Gram-negative bacteria, can be used as a highly immunogenic carrier of M2e [12,13,14]. Flagellin can bind Toll-like receptor 5 (TLR5), activating the effectors of innate immunity [15].

Lipoproteins of pathogenic bacteria are recognized by the host’s immune system as pathogen-associated molecular patterns (PAMP) through their binding to Toll-like receptor 2 (TLR2) located on the surface of antigen-presenting cells (APCs) such as macrophages and dendritic cells [16]. This binding triggers a chain of processes in the APCs, and as a result, the synthesis of cytokines released into the intercellular space occurs. Proinflammatory processes in the body are triggered, and an immune response is activated. The presence of the lipid moiety is critical for binding to TLR2 receptors [17,18]. Lipohexapeptide has been shown to be a more effective stimulator of interferon γ synthesis than the lipoprotein of *Borrelia burgdorferi* itself [18].

Similarly, the surface lipoprotein Ag473 from *Neisseria meningitidis* is able to enhance the immune response [19]. Viral proteins fused to the N-terminus of Ag473 lipopeptides have self-adjuvant properties [20]. Such fusion proteins, consisting of the target recombinant protein and a lipopeptide, ensure the delivery and uptake of both components by APCs [21], which contributes to an optimal immune response. This allows a reduction in the dose of antigens during vaccination, which reduces the risk of side effects and increases the cost-effectiveness of manufacturing.

The application of lipidation for enhancement of the immunogenicity of M2e has been reported previously [22,23]. Lipidated forms of full-size M2e (or its shorter variant comprising the first 15 amino acid residues) in which the TLR2 agonist, S-[2,3-bis(palmitoyloxy)propyl] cysteine (Pam2Cys), was attached to either the N- or C-terminus of M2e were obtained [22,23]. Animal study results show that each of these lipopeptides induced strong M2e-specific antibody responses in the absence of additional adjuvants and protected against lethal influenza [23]. All these proteins were obtained by chemical synthesis, followed by attachment of the Pam2Cys group.

In this work, we used another approach to obtain lipidated M2e derivatives. We constructed a series of recombinant proteins containing M2e peptides of the influenza A virus and the N-terminal part of the Ag473 lipopeptide from *N. meningitidis* in different combinations and produced them in *Escherichia coli*. We demonstrated that fusion to the lipopeptide greatly enhances the immunogenicity of M2e, thus, opening a new approach for the development of a “universal” influenza A vaccine.

## 2. Materials and Methods

### 2.1. Genes for Recombinant Fusion Proteins and Expression Vectors

Plasmid pQE30 was used to construct all vectors for the expression of recombinant fusion proteins. Synthetic sequence coding for four tandem copies of human consensus M2e peptide, with two endogenous cysteines mutated to serines (SLLTEVETPI RNEWGCRCND SSD, M2eh), was taken from previously constructed vector pQE60-HBc/4M2eh [11]. DNA sequences coding for a glycine-rich linker (GSGTSGSSGSGSGGSGSGGGG, 19S), alpha-helical linker (EAAAKEAAAK EAAAKEAAAK EAAAKAAA, Sp) and lipopeptide from *N. meningitidis* (LMKKLLIAAM MAAALAACSQ EAKQEVREAV QAVESDVKDT AV) were synthesized in vitro. These genetic elements were cloned in the desired combinations between the BamHI and HindIII sites of plasmid pQE30, using standard molecular cloning techniques to make 10 expression vectors, as described in the Results section.

### 2.2. Expression of Recombinant Proteins

For the expression of recombinant proteins, the corresponding vectors were introduced into *E. coli* strain DLT1270. The strains were grown with shaking in lysogeny broth (LB) or 2× tryptone yeast broth (TY) at 37 °C until the midpoint of logarithmic growth (OD_600_ ~0.5). Then, IPTG was added (to 0.5 or 1 mM concentrations in different experiments) to induce the expression of recombinant proteins. The cultures obtained in LB and 2× TY were further grown for 12–14 h at 28 °C or for 4 h at 37 °C. The optimal conditions for each of the successfully expressed proteins are shown in Table 1.

### 2.3. Purification of Recombinant Proteins

After induction, cells were collected by centrifugation (4000× *g*, 30 min) and resuspended in Tris-phosphate buffer (30 mM Tris-OH, 30 mM NaH_2_PO_4_, pH 8.0) with 500 mM NaCl and 20 mM imidazole. The suspension was treated with 1 mg/mL lysozyme for 15 min at room temperature, followed by freezing at −20 °C. The suspension was thawed and subjected to sonication (Bandelin SONOPULS HD 2200, cycle 10%, 30 s), followed by centrifugation at 13,000× *g* for 5 min. The supernatant and pellet were analyzed by SDS–PAGE, revealing the distribution of the target protein between the soluble and insoluble fractions.

For purification of soluble proteins, the supernatant was applied to Ni-NTA agarose (Qiagen, Hilden, Germany), equilibrated with Tris-phosphate buffer containing 500 mM NaCl and 20 mM imidazole and incubated for 30 min with gentle shaking. The unbound proteins were removed by washing the resin with Tris-phosphate buffer containing 500 mM NaCl and 40 mM imidazole. The recombinant protein was eluted with Tris-phosphate buffer containing 250 mM NaCl and 1 M imidazole and dialyzed against 10 mM Na-phosphate buffer with 150 mM NaCl (pH 7.4).

The pellet obtained upon centrifugation of lysed cells was used for purification of insoluble proteins. The pellet was resuspended in Tris-phosphate buffer containing 500 mM NaCl, 20 mM imidazole and 7 M guanidine chloride. The suspension was treated by ultrasonication and incubated at room temperature for 15 min, followed by centrifugation at 13,000× *g* for 10 min. The supernatant was applied to Ni-NTA agarose, equilibrated with Tris-phosphate buffer containing 500 mM NaCl and 20 mM imidazole and incubated for 30 min with gentle shaking. Following the binding of the target protein, the resin was washed first with Tris-phosphate buffer containing 500 mM NaCl, 40 mM imidazole and 7 M guanidine chloride, followed by three washes with the same buffer containing 9 M urea instead of guanidine chloride. The recombinant protein was eluted with Tris-phosphate buffer containing 250 mM NaCl, 1 M imidazole and 4.5 M urea. The recombinant protein was dialyzed against 10 mM Na-phosphate buffer with 2 M urea. This was followed by sequential dialysis against the same buffer containing 1 M of urea, 0.5 M of urea, and, finally, against the same buffer without urea.

### 2.4. Western Blotting

The protein samples were subjected to SDS–PAGE in a 10% (*w/v*) gel. After electrophoresis, the proteins were transferred from the gel onto a Hybond-P membrane (GE Healthcare, Chicago, IL, USA) by semi-dry transfer using the Trans-Blot Turbo Transfer System (Bio-Rad Laboratories, Waltham, MA, USA). Each sample was treated with a 5% (*w*/*v*) solution of casein in phosphate-buffered saline (PBS) to prevent unspecific binding of antibodies to the membrane. Then, the membrane was probed with mouse monoclonal antibodies, specific for the M2e peptide and, subsequently, incubated with anti-mouse IgG HRP conjugate (W4021, Promega, Madison, WI, USA). Specific protein complexes were visualized using a Western Blot ECL Plus kit (GE Healthcare).

### 2.5. Electron and Atomic Force Microscopy

Purified proteins were examined in a transmission electron microscope, JEM 100CXII (JEOL, Tokyo, Japan). For atomic force microscopy, an Integra Prima microscope and Nova SPM software (NT-MDT, Moscow, Russia) were used. Scanning was performed in semicontact mode using a 35 nm gold cantilever, NSG01 (NT-MDT). The protein sample was applied to a sapphire substrate, coated with mica and dried at room temperature. PBS was used as a negative control.

### 2.6. Immunization of Mice

Female BALB/c mice (16–18 g) were immunized subcutaneously in the front of the back three times at two-week intervals with 50 µg of protein without adjuvants. The control group was injected with PBS. Five mice were used for each group in the experiment. Blood samples were collected from the immunized animals on day 14, after the third immunization.

The study was carried out in accordance with the Russian Guidelines for the Care and Use of Laboratory Animals. The protocol was approved by the Committee for Ethics of Animal Experimentation at the Research Institute of Influenza (Permit ID 13/a, approved 21.10.2019).

### 2.7. Antibody Detection in the Sera

M2e-specific antibody titers in sera of individual immunized mice were determined by an enzyme-linked immunosorbent assay (ELISA), essentially as previously described [24]. The synthetic peptide G37 (SLLTEVETPIRNEWGCRCNDSSD), corresponding to the M2e consensus sequence of human influenza A viruses, was used to coat the ELISA plates. As a conjugate, goat monoclonal anti-mouse IgG (Abcam, Cambridge, UK) labeled with horseradish peroxidase was used. Differences between antibody levels were evaluated by the Mann–Whitney U test. If a *p* value was less than 0.05, the difference was considered significant.

## 3. Results

### 3.1. Design of Recombinant Proteins, Expression and Purification

Since an increase in the number of M2e peptides in the recombinant protein enhances the immune response against M2e [11,25,26], we included four or eight tandem copies of M2e in the candidate vaccine proteins. The two cysteine residues in M2e were changed to serines to prevent aggregation due to the formation of disulfide bonds under oxidative conditions; such modification does not alter the immunogenic properties of M2e [26]. To obtain lipidated proteins, we included a 42-amino-acid-long lipopeptide from *N. meningitidis*. Either a single copy of the lipopeptide upstream of M2e peptides or two copies flanking M2e were introduced. Lipopeptides and M2e repeats in some constructs were separated by a “hard” 28-amino-acid-long alpha-helical linker to facilitate appropriate folding of the recombinant proteins [27]. Control proteins comprised four or eight copies of M2e, flanked by alpha-helical linkers, but did not contain lipopeptides.

**Figure 1:**
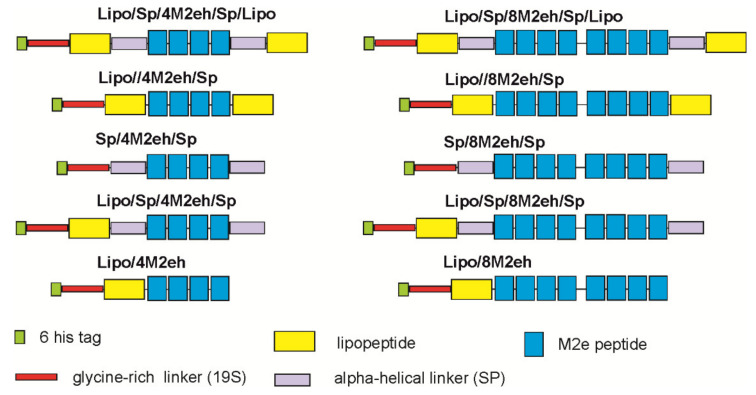
Schematic representation of genetic fusion constructs.

All fusion genes were cloned in the plasmid pQE30, enabling the expression of proteins with an N-terminal hexahistidine tag. In order to prevent possible spatial tag hiding in the fusion proteins, we introduced flexible 19-amino-acid-long glycine–serine linkers [28] at the junction points between the tag and the rest of the proteins. Altogether, 10 genes coding for M2e fusion proteins were constructed (Figure 1) and expressed in *E. coli*.

**Figure 2:**
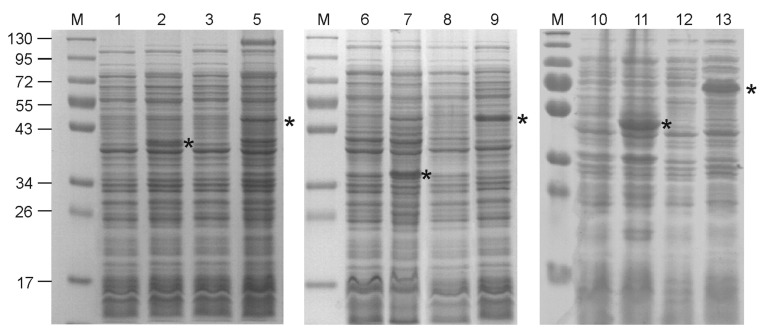
Expression of the recombinant proteins in *E. coli*. Protein samples were analyzed by SDS–PAGE. M—molecular weight markers (sizes shown in kD). Lanes 1, 3, 6, 8, 10 and 12 are proteins from *E. coli* strains producing Lipo/4M2eh/Lipo, Lipo/8M2eh/Lipo, Sp/4M2eh/Sp, Sp/8M2eh/Sp, Lipo/Sp/4M2eh/Sp/Lipo and Lipo/Sp/8M2eh/Sp/Lipo, respectively, before induction. Lanes 2, 5, 7, 9, 11 and 13 are proteins from the same strains as in lanes 1, 3, 6, 8, 10 and 12, upon induction. The positions of the target proteins are shown with asterisks (*).

Interestingly, the expression of all four hybrid proteins containing single copies of lipopeptide was unsuccessful under all tested conditions due to the arrest of culture growth upon the addition of the inducer and subsequent cell lysis. Further, all hybrid proteins with two copies of the lipopeptide, as well as control proteins without lipopeptides, were successfully produced (Figure 2). The inclusion of alpha-helical linkers between M2e and lipopeptides considerably improved expression levels (Figure 2). Recombinant proteins containing lipopeptides appeared to be mostly insoluble, while control proteins Sp/4M2eh/Sp and Sp/8M2eh/Sp occurred in soluble fractions.

**Figure 3:**
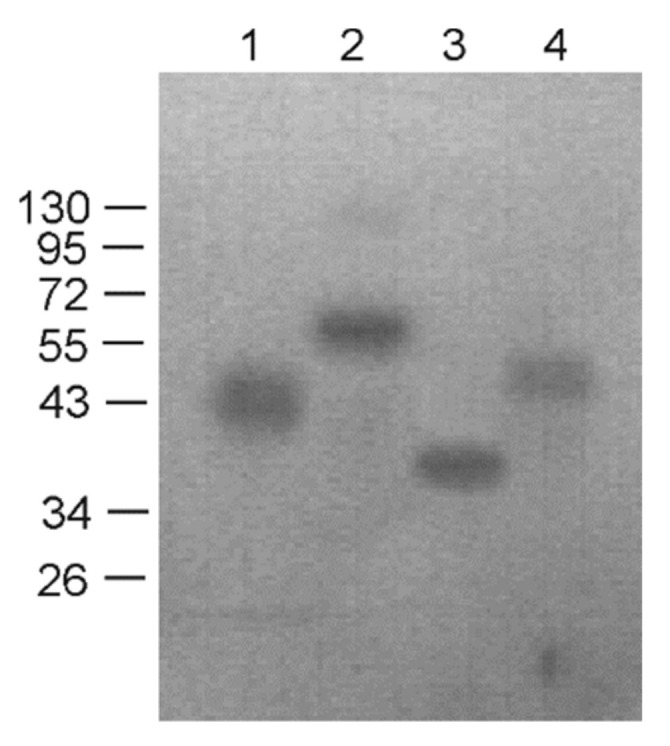
Western blot analysis of purified recombinant proteins with antibodies against M2eh. Lanes 1–4 are purified proteins Lipo/Sp/4M2eh/Sp/Lipo, Lipo/Sp/8M2eh/Sp/Lipo, Sp/4M2eh/Sp and Sp/8M2eh/Sp, respectively. Positions and sizes (in kD) of molecular weight markers are shown on the left.

All subsequent experiments on expression and purification were performed for the well-expressed fusion proteins Lipo/Sp/4M2eh/Sp/Lipo and Lipo/Sp/8M2eh/Sp/Lipo and appropriate controls without lipopeptides. The recombinant proteins carrying an N-terminal six-histidine tag were purified using nickel-affinity chromatography under denaturing conditions and then dialyzed against PBS. The obtained proteins remained soluble after dialysis. The purified recombinant proteins were identified by Western blot analysis using anti-M2e antibodies (Figure 3).

### 3.2. Recombinant Proteins Containing Lipopeptides Form Virus-Like Particles

The formation of nanosized structures significantly increases the immunogenicity of corresponding proteins [29]. Therefore, purified recombinant proteins after refolding were analyzed for the presence of nanosized particles, using atomic force and electron microscopies.

**Figure 4:**
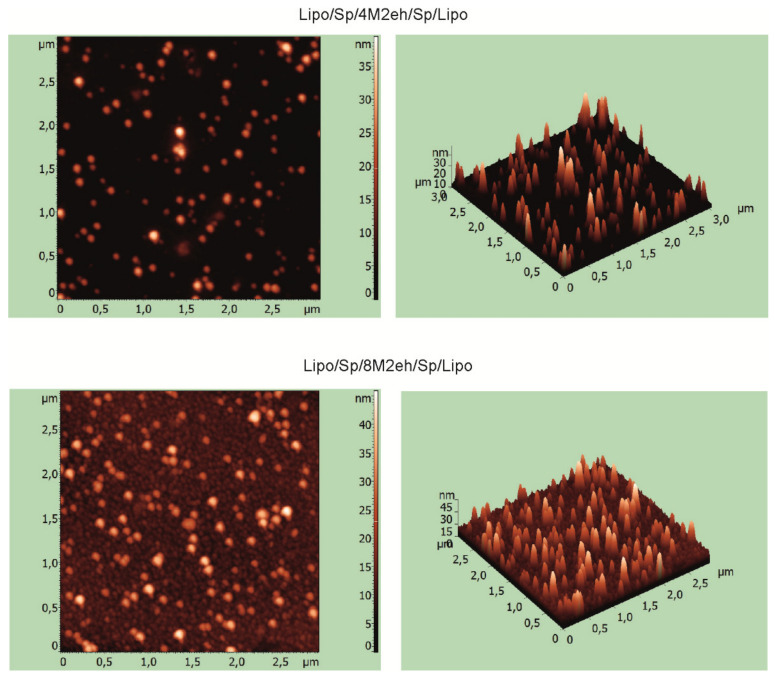
Atomic force microscopy analysis of purified recombinant proteins Lipo/Sp/4M2eh/Sp/Lipo and Lipo/Sp/8M2eh/Sp/Lipo.

**Figure 5:**
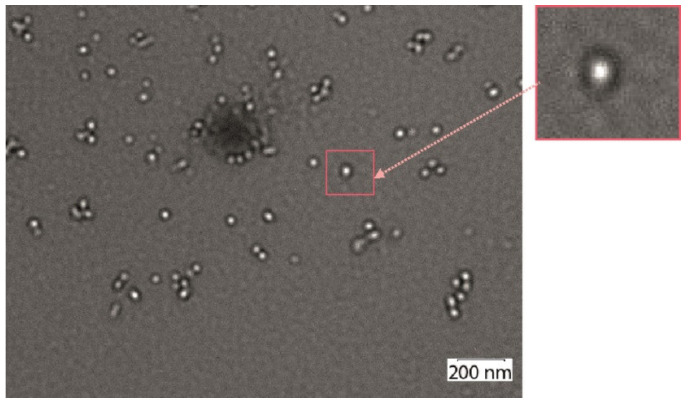
Electron microscopic analysis of virus-like particles formed by Lipo/Sp/8M2eh/Sp/Lipoprotein. The upper left part shows the structure of one of the particles on a larger scale.

AFM results indicated the proteins Lipo/Sp/4M2eh/Sp/Lipo and Lipo/Sp/8M2eh/Sp/Lipo form particles with sizes of 20–35 and 30–40 nm, respectively (Figure 4). Nanosized particles were not observed in samples of control proteins Sp/4M2eh/Sp and Sp/8M2eh/Sp. Analysis of the Lipo/Sp/8M2eh/Sp/Lipo sample using transmission electron microscopy revealed spherical particles, with sizes between 20 and 30 nm and electronically transparent cores (Figure 5).

### 3.3. The Presence of Lipopeptide Increases Immunogenicity of the Fusion Proteins

**Figure 6:**
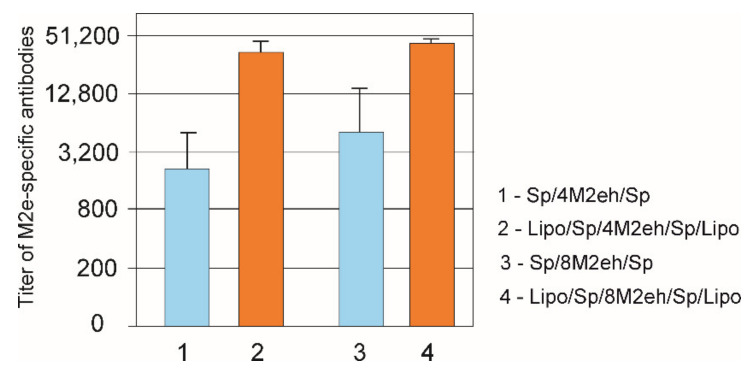
Titers of anti-M2e IgG antibodies in sera of mice immunized with proteins Sp/4M2eh/Sp, Lipo/Sp/4M2eh/Sp/Lipo, Sp/8M2eh/Sp and Lipo/Sp/8M2eh/Sp/Lipo. The results are expressed as the geometric mean titer ± the standard deviation for each group.

Mice were immunized with purified proteins Lipo/Sp/4M2eh/Sp/Lipo, Lipo/Sp/8M2eh/Sp/Lipo, Sp/4M2eh/Sp and Sp/8M2eh/Sp without additional adjuvants to characterize the immunogenicity of the fusion proteins. We analyzed the sera by ELISA to identify IgG antibodies raised against M2e (Figure 6). A strong immune response developed in mice immunized with Lipo/Sp/8M2eh/Sp/Lipo and Lipo/Sp/4M2eh/Sp/Lipoproteins. The control proteins lacking the lipopeptides were much less immunogenic (*p* < 0.05). Higher levels of anti-Me antibodies were detected for proteins containing eight copies of M2e than for their counterparts with four copies. However, differences in the antibody levels within these two pairs were not statistically significant.

## 4. Discussion

Modern peptide and recombinant vaccines are considered to be safer and have fewer side effects than traditional inactivated and live attenuated vaccines. However, such vaccines are often weakly immunogenic and require the use of adjuvants. Lipopeptides can be used as adjuvants since they are recognized by the immune system as pathogen-specific signals, inducing an immune response through binding to TLR2 [16,17,18]. In this work, we used a peptide derived from the surface lipoprotein Ag473 of *N. meningitides* containing the lipidation site as an internal adjuvant. Antigens fused to the N-terminus of this lipopeptide exhibited self-adjuvant properties [20]. In one series of recombinant proteins, the lipopeptide was included at the N-terminus of the antigen. In a second, for the introduction of additional lipid moiety, the lipopeptide was also included at the C-terminus (Figure 1).

In order to develop a broad-specificity recombinant vaccine against influenza, we used the M2e peptide as an antigen. In fusion proteins, an increase in the number of copies of the M2e peptide from one to four has been shown to significantly enhance the immune response [11,26]. Therefore, in order to enhance the immune response towards M2e and evaluate the dose-effect, we obtained recombinant proteins containing four or eight copies of the M2e peptide. In some fusion proteins, the M2e tandem copies were separated from the lipopeptides by alpha-helical linkers. This linker, (EAAAK) × 5, can form α-helices stabilized by Glu–Lys salt bridges [30]. This α-helix is a rigid structure stabilized due to hydrogen bonds. Compared to flexible linkers, helical linkers can efficiently spatially separate different functional domains [31].

The constructed proteins were compared in terms of expression efficiency in *E. coli*, solubility, ability to form nanosized particles and immunogenicity. Control proteins without lipopeptides, Sp/4M2eh/Sp and Sp/8M2eh/Sp were successfully expressed in soluble forms. In contrast, the expression levels of Lipo/Sp/4M2eh/Sp/Lipo and Lipo/Sp/8M2eh/Sp/Lipo were lower, and the proteins appeared in an insoluble fraction. Such a pattern was expected if we take into account the hydrophobic nature of the lipidated peptides. Nevertheless, both proteins were obtained in a soluble form upon refolding through sequential dialysis against decreasing concentrations of urea.

Rather unexpectedly, upon refolding, the Lipo/Sp/4M2eh/Sp/Lipo and Lipo/Sp/8M2eh/Sp/Lipoproteins formed particles 20–35 and 20–60 nm in size, respectively, with an electron-transparent core. Such a structure may reflect the localization of lipopeptides inside the particles and the remaining hydrophilic part of the protein forming an outer shell [23,32]. Due to the lipid tail attached to the hydrophilic peptide, such recombinant proteins have amphiphilic properties. Lipid tails promote self-assembly of nanoparticles, such as micelles and fibrils, with a nucleus composed of lipid fragments, while the epitopes become directed outward.

The presence of α-helices could drive protein–protein interactions, leading to oligomerization of these proteins [33]. Nanoparticles could also be formed due to the coiled-coil interaction of helices within the heptad repeat (IEKKIEA) × 4 in combination with synthetic lipopeptides [34]. Such particles had hydrophobic cores, while the target epitopes were exposed on their surface; they induced strong epitope-specific humoral immune responses in mice without the use of an additional adjuvant [34]. The particles obtained in this work probably have a similar structure.

Interestingly, recombinant proteins containing only a single copy of lipopeptide appeared to be toxic to *E. coli* cells, as evidenced by an arrest of cell growth upon the induction of expression. Presumably, proteins with only one lipidation site cannot form particulate structures in which attached lipids are hidden in the interior, as this occurred in the case of fusion proteins with two copies of lipopeptides at the termini.

Animal experiments demonstrated high immunogenicity of the obtained nanoparticles. Three-fold subcutaneous immunization of mice with lipidated fusion proteins induced much higher titers of anti-M2e antibodies compared to immunization with similar proteins without lipopeptides. The influence of the number of M2e copies in the fusion protein on the immunogenicity was rather limited. Although mice immunized with proteins comprising eight M2e peptides received twice the molar concentration of M2e, they induced only slightly higher anti-M2e response than their equivalents with four copies, and the differences in antibody titers were not statistically significant. The immunogenicity and protective activity of HBc particles carrying M2e peptides correlated with the increase of the number of M2e copies in the hybrid protein from one to four. The higher immune response induced by HBc/4M2e particles was not simply due to the dose of M2e peptide, but, rather, reflected enhanced immunogenicity of the particles [11]. The particles obtained in this work likely have a different structure with a limited number of M2e peptides exposed on the surface.

The levels of anti-M2e antibodies in sera of mice immunized with Lipo/Sp/4M2eh/Sp/Lipo and Lipo/Sp/8M2eh/Sp/Lipoproteins were in the same range (10^4^ to 10^6^) as in studies in which candidate M2e-based vaccines were shown to confer a high level of protection against lethal influenza challenge (e.g., [5,23,24,26,35,36,37,38]). Therefore, although the challenge experiment was beyond the scope of this work, we anticipate that our proteins will exhibit good protective properties.

Previously, self-adjuvating vaccine candidates based on a lipidated form of M2e were obtained by chemical synthesis [23]. Although these proteins induced strong M2e-specific antibody responses, inherent limitations of chemical synthesis capabilities did not allow for efficient production of proteins containing several copies of M2e and other functionally important components. The inclusion of α-helical linkers and lipopeptides into the fusion protein enabled the formation of nanosized particles, known to increase the immunogenicity of the corresponding antigens. We expect the use of a simple and inexpensive bacterial expression system will expand the possibilities for introducing various adjuvant components to M2e, which will allow the creation of effective candidate influenza vaccines in the future.

## 5. Conclusions

In conclusion, we reported the construction of several variants of recombinant fusion proteins based on the M2e peptide of the human influenza A virus containing adjuvant compounds. Addition of alpha-helical linkers and *N. meningitidis* lipopeptide sequences at the N- and C-termini of four or eight tandem copies of M2e peptide enabled the formation of nanosized particles and strongly enhanced the immune response against M2e in immunized mice.

## Figures and Tables

**Table 1:** Conditions used for the expression of recombinant proteins.

Protein	Growth Medium	Induction Conditions for Expression of the Target Gene		
		Time (h)	Temperature	IPTG
Lipo/Sp/4M2eh/Sp/Lipo	2× TY	4 h	37 °C	0.5 mM
Lipo/Sp/8M2eh/Sp/Lipo	2× TY	4 h	37 °C	0.5 mM
Lipo/4M2eh/Lipo	2× TY	4 h	37 °C	0.5 mM
Lipo/8M2eh/Lipo	2× TY	4 h	37 °C	0.5 mM
Sp/4M2eh/Sp	LB	12–14 h	28 °C	1 mM
Sp/8M2eh/Sp	LB	12–14 h	28 °C	1 mM
